# 3D Cartilage Regeneration With Certain Shape and Mechanical Strength Based on Engineered Cartilage Gel and Decalcified Bone Matrix

**DOI:** 10.3389/fcell.2021.638115

**Published:** 2021-02-26

**Authors:** Zheng Ci, Ying Zhang, Yahui Wang, Gaoyang Wu, Mengjie Hou, Peiling Zhang, Litao Jia, Baoshuai Bai, Yilin Cao, Yu Liu, Guangdong Zhou

**Affiliations:** ^1^Research Institute of Plastic Surgery, Wei Fang Medical College, Wei Fang, China; ^2^Shanghai Key Laboratory of Tissue Engineering, Department of Plastic and Reconstructive Surgery, Shanghai Ninth People’s Hospital, Shanghai Jiao Tong University School of Medicine, Shanghai, China; ^3^National Tissue Engineering Center of China, Shanghai, China; ^4^Plastic Surgery Hospital, Chinese Academy of Medical Sciences and Peking Union Medical College, Beijing, China

**Keywords:** 3D cartilage regeneration, engineered cartilage gel, decalcified bone matrix, tissue engineering, nutrient efficiency

## Abstract

Scaffold-free cartilage-sheet technology can stably regenerate high-quality cartilage tissue *in vivo*. However, uncontrolled shape maintenance and mechanical strength greatly hinder its clinical translation. Decalcified bone matrix (DBM) has high porosity, a suitable pore structure, and good biocompatibility, as well as controlled shape and mechanical strength. In this study, cartilage sheet was prepared into engineered cartilage gel (ECG) and combined with DBM to explore the feasibility of regenerating 3D cartilage with controlled shape and mechanical strength. The results indicated that ECG cultured *in vitro* for 3 days (3 d) and 15 days (15 d) showed good biocompatibility with DBM, and the ECG–DBM constructs successfully regenerated viable 3D cartilage with typical mature cartilage features in both nude mice and autologous goats. Additionally, the regenerated cartilage had comparable mechanical properties to native cartilage and maintained its original shape. To further determine the optimal seeding parameters for ECG, the 3 d ECG regenerated using human chondrocytes was diluted in different concentrations (1:3, 1:2, and 1:1) for seeding and *in vivo* implantation. The results showed that the regenerated cartilage in the 1:2 group exhibited better shape maintenance and homogeneity than the other groups. The current study established a novel mode of 3D cartilage regeneration based on the design concept of steel (DBM)-reinforced concrete (ECG) and successfully regenerated homogenous and mature 3D cartilage with controlled shape and mechanical strength, which hopefully provides an ideal cartilage graft for the repair of various cartilage defects.

## Introduction

The clinical treatment of cartilage defects is a significant challenge (Gomez et al., 2020; Mardones et al., 2020; Pattappa et al., 2020), as current methods do not provide an ideal graft for repairing the cartilage defects. Autologous cartilage transplantation is currently the most effective method for treating cartilage defects; however, there is limited source tissue, and the potential exists for irreversible damage to the donor area (Schinhan et al., 2013; Ruta et al., 2016). Tissue engineering provides a new approach for treating various cartilage defects and can be used to regenerate sufficient autologous viable cartilage tissue during *in vitro* proliferation with a small number of chondrocytes and minimal trauma (Zheng et al., 2014; He et al., 2018; Zhou et al., 2018).

Various animal and clinical experiments have demonstrated that scaffold-free cartilage-sheet technologies can stably regenerate high-quality cartilage tissue *in vivo* (Li et al., 2017, 2019; He et al., 2018). Our latest studies demonstrated that engineered cartilage gel (ECG) produced by cartilage-sheet technology have excellent ability to regenerate cartilage (unpublished data). However, the application of ECG is mainly limited to minimally invasive fillings, which are not suitable for repairing cartilage defects with specific shape and strength requirements because ECG cannot be shaped, ECG does not provide adequate mechanical strength, and ECG particles are too large to seed in conventional tissue regeneration scaffolds that have a relatively small pore size.

The above limitations greatly hinder the clinical application of ECG. A suitable framework with controlled shape, mechanical strength, and fine pore size is required to address these problems. Current scaffolds suitable for cell seeding cannot meet these requirements; therefore, here, we introduce DBM as a framework for ECG.

DBM is an ideal natural tissue regeneration scaffold with biocompatibility and immunogenicity, controllable shape, and mechanical strength that can be controlled by the degree of decalcification (Zheng et al., 2004; Liese et al., 2013; Meng et al., 2015; Xie et al., 2017). However, because of its large pore size and the difficulty in controlling its uniformity, DBM readily loses cells after seeding and cannot be directly used for cartilage regeneration (Man et al., 2016; Xu B. et al., 2017). Additionally, because of its rapid degradation (ten Koppel et al., 1998; van Osch et al., 1999), DBM cannot be used alone as a tissue regeneration scaffold, which greatly limits its application in the field of cartilage regeneration. However, the large pore size and controllable mechanical strength of DBM are precisely suitable for seeding ECG, which has a large particle size. Therefore, the current study investigated a novel mode of 3D cartilage regeneration based on the design concept of steel (DBM)-reinforced concrete (ECG). Although the combination of ECG and DBM can overcome their individual limitations and take full advantage of both materials, there are no reports on whether the steel (DBM)-reinforced concrete (ECG) concept can achieve 3D cartilage regeneration with controlled shape and mechanical strength. To prove the steel (DBM)-reinforced concrete (ECG) concept, the following scientific questions must be answered. (1) Can cartilage regeneration be realized using this concept? (2) Which mature stage of ECG is better for seeding DBM for cartilage regeneration? (3) Does the ECG need to be diluted, and if so, what proportion is optimal? These parameters not only affect the feasibility of the future clinical translation of ECG–DBM constructs and but also play an important role in the efficiency of cartilage regeneration *in vivo*.

To address these questions, goat chondrocyte suspensions with a high density were seeded in six-well cell culture plates and cultured for 3 days (3 d) to produce early ECG or 15 days (15 d) for mature ECG. The ECG–DBM constructs were formed by direct seeding (early ECG) or centrifuging (mature ECG) and implanted into nude mice and autologous goats to verify the feasibility and the proper parameter of cartilage regeneration. Furthermore, human chondrocyte ECG was suspended in gradient dilutions, seeded in DBM, and implanted into nude mice to determine whether the steel (DBM)-reinforced concrete (ECG) concept is suitable for human cartilage regeneration. The optimal ECG dilution for cartilage regeneration efficiency in future clinical applications was also evaluated.

## Materials and Methods

### Animals

Three 6-month-old goats (Shanghai Jiagan Biological Technology Co., Shanghai, China) were used in this study. All animal experiments were approved by the Weifang Medical College Ethics Committee.

### Isolation and Culture of Goat Chondrocytes

Auricular cartilage was obtained from autologous goat and cut into 1.0 mm pieces, washed using phosphate-buffered solution (PBS), and digested using 0.15% collagenase (Worthington Biochemical Corp., NJ, United States) for isolating chondrocytes, as previously reported (Rodriguez et al., 1999). Isolated chondrocytes were cultured in Dulbecco’s modified Eagle’s medium (DMEM) medium (Gibco BRL, Grand Island, NY, United States) containing 10% fetal bovine serum (Gibco BRL) and 1% antibiotic-antimycotic (Gibco BRL). Passage 2 chondrocytes were used to form cartilage sheets (Li et al., 2019).

### Formation of Engineered Cartilage Gel

Cartilage sheets were prepared as previously established (Xue et al., 2018). Goat chondrocytes in the second passage were suspended and seeded in six-well cell culture plates at a density of 1.5 × 10^7^ cells/well. The chondrocytes were then cultured in chondrogenic medium comprising DMEM, 10 ng/ml of TGF-β1 (R&D Systems Inc. Minneapolis, MN, United States), 40 ng/ml of dexamethasone (Sigma-Aldrich, St. Louis, MO, United States), 100 ng/ml of IGF-I (R&D Systems Inc. Minneapolis, MN, United States), 1% insulin-transferrin-selenium-linoleic acid (ITS, ScienCell, CA, United States), and 1% antibiotic-antimycotic (Gibco BRL) for 3 and 15 days. The 3 d ECG was prepared from 3 days cartilage sheets that were collected directly using a 5 ml syringe and mixed well with the 1.5-fold chondrogenic medium. The 15 d ECG was prepared from 15 days cartilage sheets that were minced into pieces and collected in a centrifuge tube without any further attenuation.

### Harvest and Culture of Human Chondrocytes

The protocols for the use of human cells were approved by the Weifang Medical College Ethics Committee. Abandoned cartilage tissue was donated by patients. To isolate the chondrocytes, the cartilage pieces were washed by PBS and digested by 0.15% collagenase (Worthington Biochemical Corp., Freehold, NJ, United States) as previously described (Deng et al., 2009). The cells were then harvested and cultured as previously reported (Deng et al., 2009). Chondrocytes from passage 2 were used to form human cartilage sheets (Li et al., 2019).

### Formation of Engineered Cartilage Gel With Different Dilution Ratios

Human cartilage sheets were prepared using a process that was similar to that used with the goat chondrocytes. Human chondrocytes from passage 2 were seeded in six-well cell culture plates at a density of 1.5 × 10^7^ cells/well. The chondrocytes were cultured in chondrogenic medium for 3 days, and the 3 days cartilage sheets were collected using a 5 ml syringe and mixed with onefold, twofold, or threefold chondrogenic medium.

### Formation of the Engineered Cartilage Gel–Decalcified Bone Matrix Constructs

DBM frameworks (Daqing Bio Co. LTD, Chongqing, China) were cut into cuboid constructs 7 mm long, 5 mm wide, and 2.5 mm thick. Different methods were used to form the chondrocyte ECG–DBM constructs. The 3 d ECG was directly seeded into the DBM and incubated for 2 h. The 15 d ECG and DBM were combined by centrifuging for 2 min at 500 rpm and then incubated for 2 h. The ECG with different dilution ratios were directly seeded into DBM and incubated for 2 h. All ECG–DBM constructs were then gently transferred to new six-well plates. After 3 days, the constructs were subcutaneously implanted into autologous goats and nude mice.

### Biocompatibility Between Engineered Cartilage Gel and Decalcified Bone Matrix and the Engineered Cartilage Gel Adhesion Efficiency

After *in vitro* culture for 24 and 72 h, the ECG–DBM constructs were washed by PBS and fixed overnight at 4°C in 0.05% glutaraldehyde. Both the DBM framework and ECG–DBM constructs were critical point dried and examined by scanning electron microscopy (SEM; Philips XL-30, Amsterdam, Netherlands) to observe the pore size distribution of the DBM framework and attachment and distribution of ECG and to assess the ECG synthesis on the DBM (Xu Y. et al., 2017). The ECG adhesion rate was determined by the ratio of the DNA content of ECG that was seeded into the DBM and the DNA content of ECG–DBM constructs that were cultured for 24 h. The DNA content of the samples (*n* = 5 per group) was quantified by a Quant-iT PicoGreen dsDNA assay (Invitrogen, Carlsbad, CA, United States) as previously described (Schagemann et al., 2010).

### Mechanical Analysis

The Young modulus of the regenerated cartilage was tested using a mechanical analyzer (Instron-5542, Instron, Canton, MA). As previously described (Liu et al., 2008), samples from different groups (*n* = 6 per group) were cut into 4 mm-diameter cylinders. A constant compressive strain at a speed of 0.5 mm/min was applied until 80% of the maximal deformation. The stress–strain curves were obtained from the first 40%. The Young modulus was calculated according to the stress–strain curves.

### Histological and Immunohistochemical Analyses

To evaluate the structure and extracellular matrix (ECM) deposition of the regenerated cartilage, hematoxylin, and eosin (HE), Safranin-O, and type II collagen (mouse anti-human type II collagen monoclonal antibody, 1:100, Santa Cruz Biotechnology, Dallas, TX), staining was performed (Liu et al., 2008). The terminal deoxynucleotidyl transferase biotin dUTP nick end labeling assay (TUNEL) for apoptosis was analyzed using a TUNEL kit (Roche, Indianapolis, IN). CD3 was analyzed using mouse anti-human CD3 monoclonal antibody (1:200 in PBS, Santa Cruz Biotechnology, Santa Cruz, CA, United States). CD68 was analyzed using mouse anti-human CD68 monoclonal antibody (1:200 in PBS, Santa Cruz). Horseradish peroxidase (HRP)-conjugated anti-mouse antibody (1:200 in PBS, Santa Cruz) was then applied as a secondary antibody.

### Quantitative Analysis

Quantitative analysis was performed as previously described (Enobakhare et al., 1996; Reddy and Enwemeka, 1996; Schagemann et al., 2010). Briefly, all samples (*n* = 5 per group) were weighed using an electronic balance. The volume of each sample (*n* = 5 per group) was measured using a water displacement method. The volume change rate was determined by the ratio of the volume of the samples that were cultured *in vitro* for 72 h and the volume of the samples after 12 weeks *in vivo* The total glycosaminoglycan (GAG) content of the samples (*n* = 5 per group) was quantified using the Alcian blue method. The total collagen content of the samples (*n* = 5 per group) was detected using a hydroxyproline assay.

### Quantitative Reverse Transcription Polymerase Chain Reaction Analysis

The total RNA was extracted from 3 d and 15 d ECGs (*n* = 3 per group), and cDNA was obtained by reverse transcription (RT) according to a previously described method (Jiang et al., 2010). RT-qPCR was performed according to the manufacturer’s protocol (Thermo Fisher Scientific, Waltham, MA, United States). The expression levels of the genes Aggrecan, COLIIA1, and Sox9 were analyzed. The housekeeping gene encoding β-actin was quantified as an internal control. Forward and reverse primer sequences are listed in Table 1. Expression levels were analyzed using the 2^–ΔΔ*CT*^ method, as previously described (Livak and Schmittgen, 2001).

**TABLE 1:** Primer sequences of related genes.

Genes	Primer sequence (5′–3′)
COL2A1	GCATTGCCTACCTGGACGAAG
	TCACAGTCTCGCCCCACTTAC
Aggrecan	CAGAGGCAACCACAACAGACA
	AGCTGGGAAGGCATAAGCATG
SOX9	AAGAACAAGCCGCACGTCAA
	CCGTTCTTCACCGACTTCCTC

### Statistical Analysis

All values were expressed as mean ± standard. A *t*-test analysis of the variance was used to determine the statistical between groups using SPSS 23 software, and a value of ^∗^*P* < 0.05 was considered statistically significant.

## Results

### Formation of the Engineered Cartilage Gel

**FIGURE 1:**
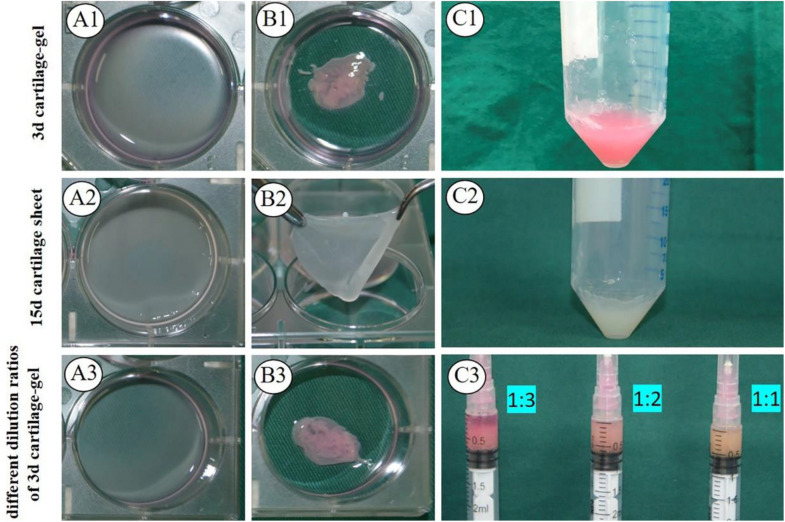
Formation of engineered cartilage gel. Gross **(A1)** and mature status **(B1)** images of 3 d goat cartilage sheet and gross images **(C1)** of 3 d engineered cartilage gel (ECG). Gross **(A2)** and mature status **(B2)** images of 15 d goat cartilage sheet and gross images **(C2)** of 15 d ECG. Gross **(A3)** and mature status **(B3)** images of a 3 d human cartilage sheet and 3 d cartilage-gel in different dilution ratios **(C3)**.

As shown in Figure 1, both types of 3 days cartilage sheet—goat chondrocyte and human chondrocyte—had liquid properties and could be collected using a 5 ml syringe (Figures 1B1,B3 and Supplementary Videos 1, 2), and the 15 days cartilage sheets were relatively tough sheets that could not be directly collected (Figure 1B2). The 3 d ECG was successfully prepared by mixing 3 days cartilage sheets with 1.5-fold medium (Figure 1C1). The 15 d ECG was prepared from 15 days cartilage sheets that were minced into pieces (Figure 1C2). And as for the expression of cartilage-related genes, 15 d ECG was higher than 3 d ECG (Supplementary Figure 1). Different dilution ECGs exhibited different behaviors: the ECG in 1:1 and 1:2 groups stably maintained a suspension, while the ECG in the 1:3 group rapidly sedimented and presented obvious stratification (Figure 1C3).

### Evaluation of the Biocompatibility Between Engineered Cartilage Gel and Decalcified Bone Matrix and the Engineered Cartilage Gel Adhesion Efficiency

**FIGURE 2:**
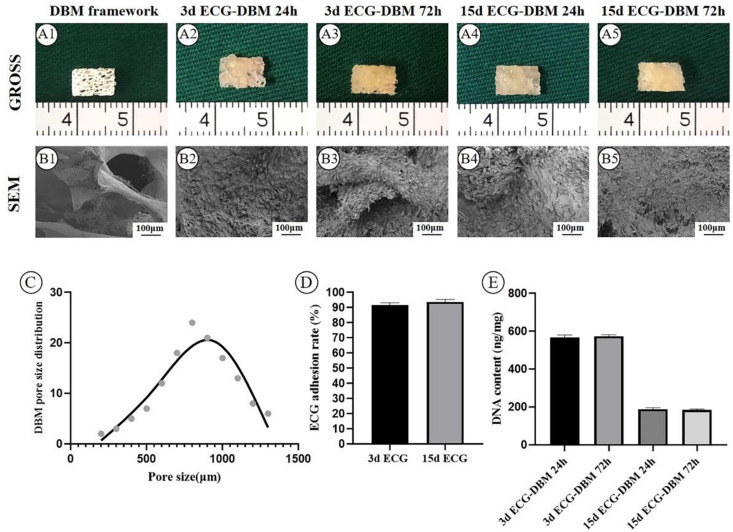
Evaluation of the biocompatibility of engineered cartilage gel (ECG) and decalcified bone matrix (DBM), and the ECG adhesion efficiency. Gross **(A1)** and SEM **(B1)** images of the DBM framework. Gross **(A2)** and SEM **(B2)** images of the 3 d ECG–DBM constructs after culture *in vitro* for 24 h. Gross **(A3)** and SEM **(B3)** images of the 3 d ECG–DBM constructs after culture *in vitro* for 72 h. Gross **(A4)** and SEM **(B4)** images of the 15 d ECG–DBM constructs after *in vitro* culture for 24 h. Gross **(A5)** and SEM **(B5)** images of the 15 d ECG–DBM constructs after *in vitro* culture for 72 h. The pore size distribution of the DBM framework **(C)**. The ECG adhesion rate of ECG **(D)**. The DNA content of the ECG–DBM framework at different times **(E)**.

The biocompatibility between ECG and DBM was evaluated using gross imaging and SEM. As shown in Figure 2, the pore size of DBM was too large and unsuitable for cell seeding (Figures 2A1,B1,C). However, both 3 d and 15 d ECGs showed biocompatibility with DBM, and the ECGs stably adhered to DBM. In the 3 days *in vitro* culture, the DBM pores of both groups were filled with ECG (Figures 2A2–A5,B2–B5). Both 3 d and 15 d ECGs had fine ECG adhesion efficiency (Figures 2D,E).

### Cartilage Regeneration *in vivo*

**FIGURE 3:**
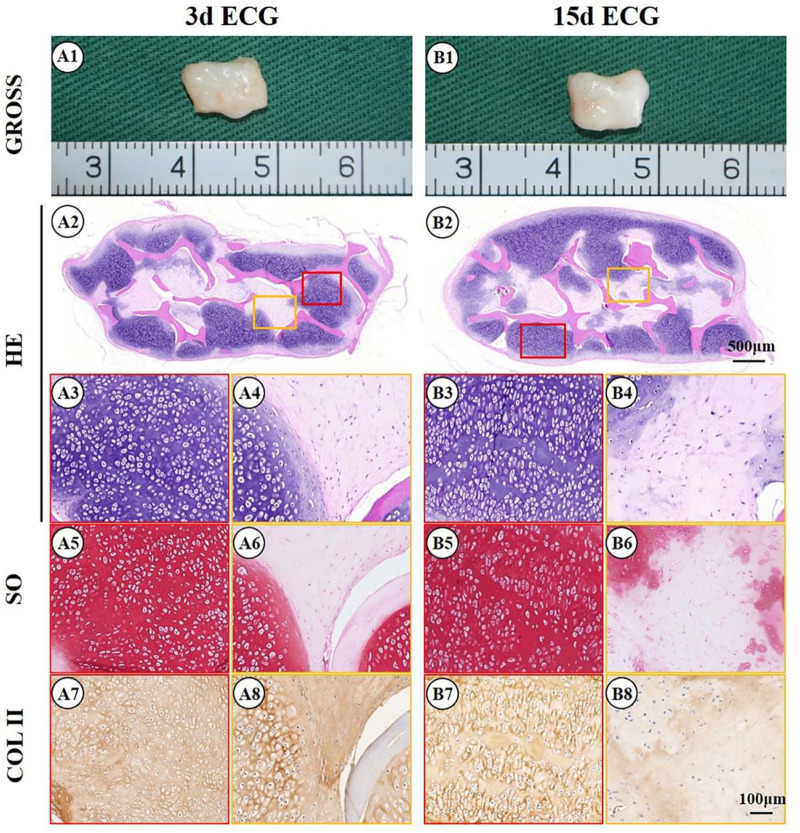
Gross view and histological examination of the cartilage regenerated by goat engineered cartilage gel (ECG) after 12 weeks *in vivo* in nude mice. Gross view, HE, Safranin-O, and collagen II immunohistochemical staining of 3 d ECG–decalcified bone matrix (DBM) **(A1–A8)** and 15 d ECG–DBM constructs **(B1–B8)**. The red box represents the mature cartilage-like tissue areas, and the orange box represents the immature cartilage-like tissue.

**FIGURE 4:**
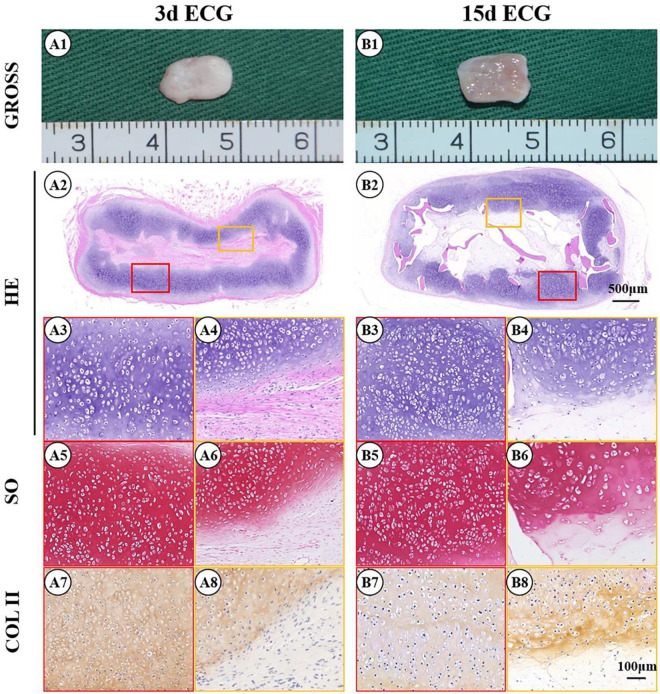
Gross view and histological examination of the cartilage regenerated by goat engineered cartilage gel (ECG) after 12 weeks *in vivo* in autologous goat. Gross view, HE, Safranin-O, and collagen II immunohistochemical staining of 3 d ECG–decalcified bone matrix (DBM) **(A1–A8)** and 15 d ECG–DBM **(B1–B8)** constructs. The red box represents the mature cartilage-like tissue areas, and the orange box represents immature cartilage-like tissue.

**FIGURE 5:**
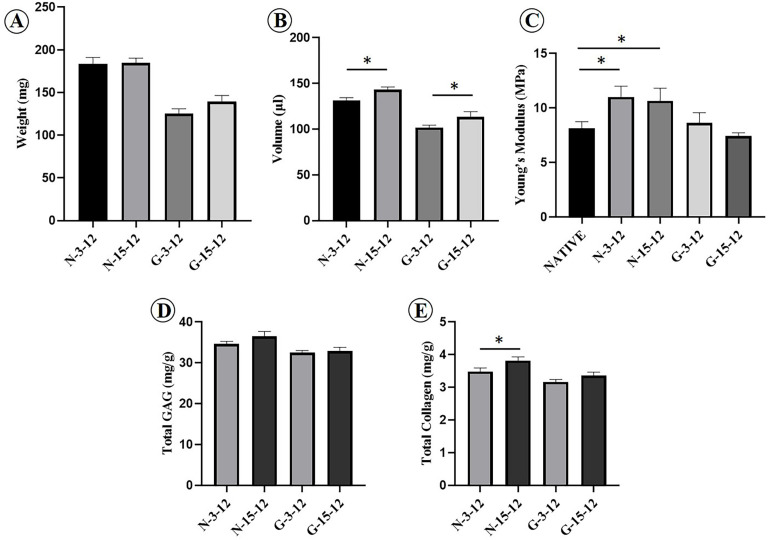
Quantitative evaluations of the cartilage regenerated by goat engineered cartilage gel (ECG) after 12 weeks *in vivo*. Quantitative analysis of the wet weight **(A)**, volume **(B)**, Young’s modulus **(C)**, total glycosaminoglycan (GAG) **(D)**, and total collagen **(E)** in different groups. N-3-12 = the cartilage regenerated by 3 d ECG–decalcified bone matrix (DBM) constructs after 12 weeks *in vivo* in nude mice; N-15-12 = the cartilage regenerated by 15 d ECG–DBM constructs after 12 weeks *in vivo* in nude mice; G-3-12 = the cartilage regenerated by 3 d ECG–DBM constructs after 12 weeks *in vivo* in autologous goats; G-15-12 = the cartilage regenerated by 15 d ECG–DBM constructs after 12 weeks *in vivo* in autologous goats. *P < 0.05.

The feasibility of cartilage regeneration using ECG–DBM constructs was the first key point in the current study. After 12 weeks, both 3 d and 15 d ECG–DBMs successfully regenerated ivory white, cartilage-like tissues that maintained their shape in both nude mouse (Figures 3A1,B1) and autologous goat models (Figures 4A1,B1). Histological analysis performed at 12 weeks showed that both the 3 d and 15 d ECM–DBM constructs formed mature cartilage-like tissue in the outer areas of the sample with abundant lacuna structure and homogenous cartilage ECM distribution in both nude mouse (Figures 3A2–A8,B2–B8) and autologous goat models (Figures 4A2–A8,B2–B8). Additionally, some immature cartilage-like tissue (15 d) and fibrous-like tissue (3 d) were observed in the inner areas of all the samples (likely because of nutrient deficiency). Consistent with the histological staining, the quantitative analysis showed that the GAG content and total collagen content and the mechanical strength of the regenerated cartilage reached favorable levels at 12 weeks for both 3 d ECG–DBM and 15 d ECG–DBM (Figure 5).

### Immuno-Inflammatory Reaction and Apoptosis

**FIGURE 6:**
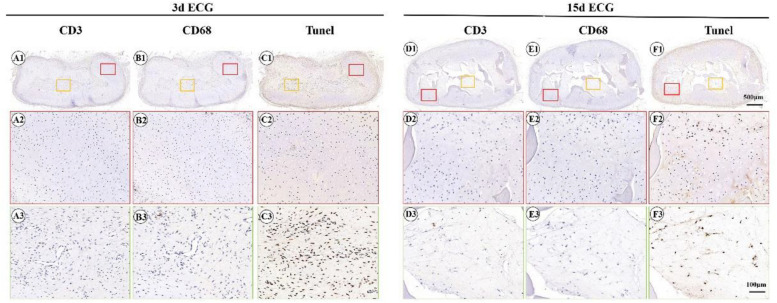
Inflammatory reactions characterized by CD3, CD68, and terminal deoxynucleotidyl transferase biotin dUTP nick end labeling assay (TUNEL) staining. CD3 staining of 3 d engineered cartilage gel–decalcified bone matrix (ECG–DBM) **(A1–A3)** and 15 d ECG–DBM constructs **(D1–D3)** after 12 weeks *in vivo* in autologous goat. CD68 staining of 3 d ECG–DBM **(B1–B3)** and 15 d ECG–DBM **(E1–E3)** constructs after 12 weeks *in vivo* in autologous goat. TUNEL staining of 3 d ECG–DBM **(C1–C3)** and 15 d ECG–DBM **(F1–F3)** constructs after 12 weeks *in vivo* in autologous goat. The red box represents the mature cartilage-like tissue areas, and the orange box represents immature cartilage-like tissue.

To further clarify the reasons for the formation of the immature cartilage/fibrous-like tissue, immunohistological examinations of CD3 (T-cell marker), CD68 (monocyte/macrophage marker), and the TUNEL assay for apoptosis were conducted. As shown in Figure 6, both the outer cartilage-like tissue and inner immature cartilage/fibrous tissue showed negative CD3 (Figures 6A1–A3,D1–D3) and CD68 expression (Figures 6B1–B3,E1–E3), which indicates that no host inflammatory cells participated in the cartilage regeneration. TUNEL staining confirmed that a large number of apoptotic cells were detected in the inner areas but not in the outer areas (Figures 6C1–C3,F1–F3), which implies that the immature cartilage/fibrous-like tissue in the inner areas probably derived from implanted ECG, whose cells were dying because of nutrient deficiency caused by the continuous cartilage-like tissue on the periphery of the implant blocking nutrient transport.

### Cartilage Regeneration of Different Dilutions of Human Engineered Cartilage Gel

**FIGURE 7:**
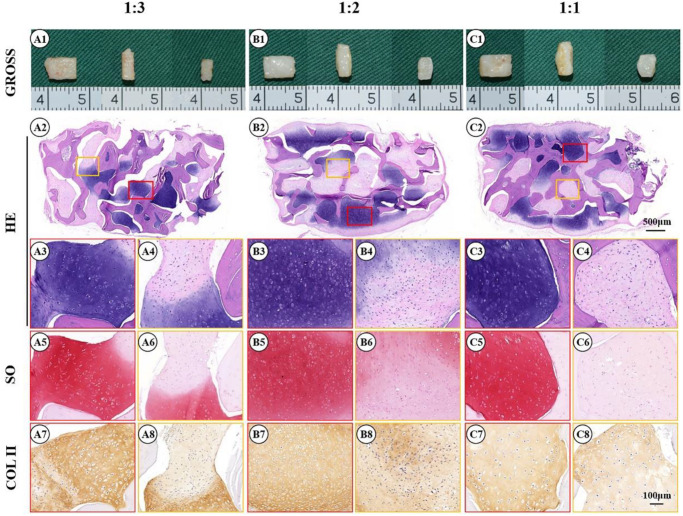
Gross view and histological examination of the cartilage regenerated by human engineered cartilage gel (ECG) after 12 weeks *in vivo* implantation in nude mouse. Gross view, HE, Safranin-O, and collagen II immunohistochemical staining of 3 d ECG–decalcified bone matrix (DBM) constructs in 1:1 **(A1–A8)**, 1:2 **(B1–B8)**, and 1:3 **(C1–C8)** dilution ratios. The red box represents the mature cartilage-like tissue areas, and the orange box represents immature cartilage-like tissue.

**FIGURE 8:**
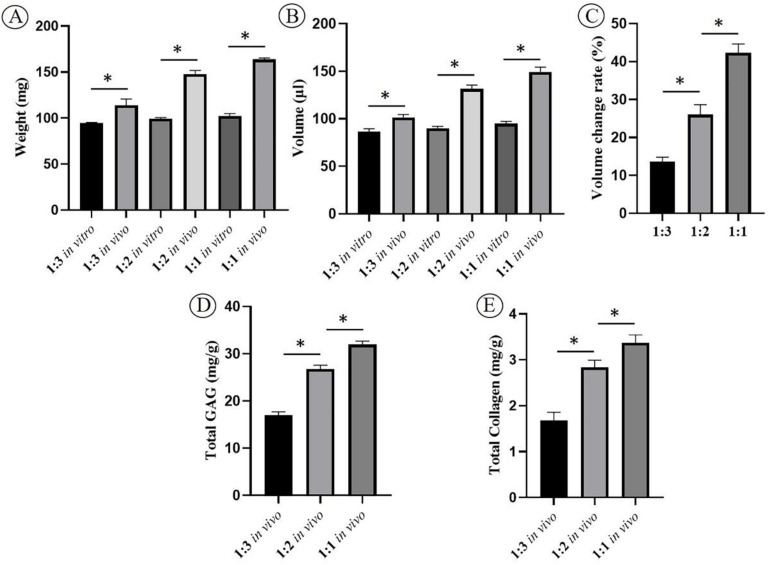
Quantitative evaluations of human engineered cartilage gel–decalcified bone matrix (ECG–DBM) constructs. Quantitative analysis of the wet weight **(A)**, volume **(B)**, and volume change rate **(C)** in different groups after 3-day culture *in vitro* (*in vitro*) and 12 weeks *in vivo* (*in vivo*). Total glycosaminoglycan (GAG) **(D)** and total collagen **(E)** in different groups after 12 weeks *in vivo* implantation (*in vivo*). 1:1 = ECG mixed well with onefold medium; 1:2 = ECG mixed well with twofold medium; 1:3 = ECG mixed well with threefold medium. *P < 0.05.

Enhancing cartilage regeneration efficiency is vital for the clinical translation of ECG. To explore the feasibility of enhancing cartilage regeneration efficiency, 3 d ECG was diluted at different ratios (1:3, 1:2, and 1:1) and combined with DBM followed by *in vivo* implantation. After 12 weeks, gross views showed that the samples in all groups formed mature cartilage-like tissues. However, the samples in the 1:1 group exhibited visible excessive proliferation and deformation compared with before implantation, while the samples in the 1:2 and 1:3 groups had relatively satisfactory shape maintenance (volume change rate < 30%, Figures 8A–C) and homogeneity of the regenerated cartilage (Figures 7A1,B1,C1, 8A–C). Histological examinations showed that the samples in the 1:2 and 1:1 groups regenerated continuous and mature cartilage-like tissue (Figures 7B2–B8,C2–C8), while only a small amount of mature cartilage-like tissue was observed in the inner areas in 1:3 group (Figures 7A2–A8). Quantitative analyses showed that all quantitative indexes, including the wet weight, volume, and cartilage ECM contents, increased significantly with decreasing dilution (Figure 8). These results indicate that an appropriate dilution is important for enhancing the cartilage regeneration efficiency and preventing excessive proliferation of the ECG–DBM constructs. In terms of efficiency, quality, homogeneity, and shape maintenance, a 1:2 dilution of ECG is appropriate for cartilage regeneration of the ECG–DBM constructs.

## Discussion

Although cartilage-sheet technology is a promising strategy for cartilage regeneration, an uncontrolled shape and poor mechanical strength greatly hinder further clinical application (Li et al., 2017, 2019). The results demonstrated that ECG produced by cartilage-sheet technology combined with DBM can regenerate 3D cartilage tissue with controlled shape and mechanical strength in both nude mice and autologous goats. Additionally, both 3 d and 15 d ECGs combined with DBM regenerated favorable cartilage tissue *in vivo*. More importantly, ECG–DBM constructed from human chondrocytes also successfully regenerated mature cartilage tissue in nude mice, and 3 days ECG at a 1:2 dilution provided the optimal conditions of those tested. The current study provides a new cartilage regeneration mode and predicts its potential in future clinical translation.

Whether ECG combined with DBM can successfully regenerate viable cartilage with a certain shape and mechanical strength is the first important issue. The current study showed that ECG of goat chondrocytes could regenerate fine cartilage tissue when combined with DBM, in both nude mice and autologous goats. Additionally, histological staining, immunohistochemical staining, and related quantification analyses confirmed that the regenerated cartilage had typical cartilage lacunae and cartilage specific ECM deposition. More importantly, these regenerated cartilages maintained their original shape and presented satisfactory mechanical strength (even greater than normal levels). These findings strongly support the concept of feasibility of steel (DBM)-reinforced concrete (ECG) to regenerate homogenous and mature 3D cartilage with controlled shape and mechanical strength. The regeneration process and principle should be as follows: At an early stage, DBM provides shape maintenance and mechanical support for cartilage regeneration. At a later stage, following DBM degradation, ECG-regenerated cartilage gradually matures and finally forms intact and continuous cartilaginous tissue, which contributes to stable shape maintenance and mechanical support. Importantly, the same results were verified using human ECG and demonstrated a high potential for future clinical applications.

After verifying the feasibility of the concept, identifying if the mature status of ECG was conducive to cartilage regeneration became the key step for future clinical application. Therefore, the cartilage tissues regenerated by short-term (3 days) and long-term (15 days) ECG–DBM constructs were systematically compared. The chondrocyte sheets cultured for 3 days were only preliminarily formed and were relatively immature. Consequently, the sheets could be directly collected to form ECG and seeded into DBM. After culture for 15 days, the formed chondrocyte sheets were mature, tough cartilage tissues could be picked up with tweezers, and they required being cut into pieces to form ECG for seeding into DBM by centrifugation. The *in vivo* results confirmed that both 3 d ECG–DBM and 15 d ECG–DBM constructs could regenerate mature cartilage tissue, and there was no significant difference, although 15 d ECG had higher expression of cartilage-related genes before seeding into the DBM construct. Nevertheless, compared with 15 d ECG, 3 d ECG had obvious advantages, including a shorter culture period, less medium requirements, easier seeding operation, higher ECG seeding efficiency, and lower contamination risk. Therefore, 3 d ECG was more suitable for the current mode of 3D cartilage regeneration.

After confirming the suitable mature status of ECG, the optimal ratio of ECG dilution was the next key parameter. To determine the effect of ECG dilution on cartilage regeneration, 3 d human ECG was diluted by gradient, and the differences in cartilage regeneration among the various dilution ratios were compared. According to the results, the samples in the 1:2 dilution group showed relatively satisfactory cartilage regeneration in terms of homogeneity, shape maintenance, and efficiency of the regenerated cartilage. In the 1:1 dilution group, although relatively mature and homogenous cartilage was successfully regenerated, the samples presented visible excessive proliferation and deformation on the surface. ECG at this dilution ratio had a high viscosity, which led to local accumulation on the surface of the construct liquidity that caused excessive proliferation and deformation on the surface. Alternatively, in the 1:3 dilution group, the samples showed heterogeneous cartilage regeneration, and mature cartilage was mainly observed in the inner areas. ECG at this dilution ratio had a relatively low viscosity, which led to ECG loss in the outer areas of the construct because of the low viscosity and low adhesion efficiency after seeding, which resulted in inferior cartilage regeneration in the outer areas. These findings suggested that appropriate ECG dilution is vital for satisfactory cartilage regeneration using ECG–DBM constructs.

Although the feasibility of the cartilage regeneration was verified using the current mode, most of samples showed inferior cartilage formation inside, which led to an obviously heterogeneous structure of the sample. The inferior cartilage formation inside was possibly caused by nutrient deficiency. After implantation *in vivo*, ECG in the outer areas of the samples easily obtained sufficient nutrients and rapidly formed a relatively mature and continuous cartilage layer, which severely blocked nutrient diffusion that led to nutrient deficiency in the inner areas and caused cell apoptosis and inferior cartilage formation (Wu et al., 2010). The results of CD3, CD68, and TUNEL staining further verified that the immature cartilage/fibrous-like tissue in the inner areas was mainly composed of apoptotic chondrocytes rather than host inflammatory cells. Alternatively, when the outer layer cartilage was discontinuous (nutrients could freely diffuse into inner areas), as shown in the 1:3 dilution group, mature cartilage was also observed in the inner areas. It is accepted that cartilage regeneration has a thickness limitation because of its avascular feature (Bonzani et al., 2006; Ge et al., 2012). Therefore, to realize homogeneity of a whole regenerated cartilage, multiple module cartilage regenerations, and a multi-layer assembly might be a feasible strategy for future clinical applications.

## Conclusion

This study established a new concept for cartilage regeneration—analogous to steel-reinforced concrete—based on ECG and DBM and demonstrated its feasibility. Additionally, the formation method and relative parameters were investigated and optimized. However, some important issues, including how to precisely control the shape and realize homogeneity of the whole regenerated cartilage, still need to be investigated. The presented results provide a new cartilage regeneration strategy with potential for future clinical translation.

## Data Availability Statement

The original contributions presented in the study are included in the article/Supplementary Material, further inquiries can be directed to the corresponding author/s.

## Ethics Statement

The studies involving human participants were reviewed and approved by the Weifang Medical College Ethics Committee. The patients/participants provided their written informed consent to participate in this study. The animal study was reviewed and approved by the Weifang Medical College Ethics Committee.

## Author Contributions

ZC, YZ, and YW: scaffolds design, culture cells, and animal operation. GW, MH, PZ, LJ, and BB: relative data analyses. YC and YL: revision. GZ: funding acquisition, review, and editing. All authors contributed to the article and approved the submitted version.

## Conflict of Interest

The authors declare that the research was conducted in the absence of any commercial or financial relationships that could be construed as a potential conflict of interest.
