# Supplementary figures and images for: 3D Cartilage Regeneration With Certain Shape and Mechanical Strength Based on Engineered Cartilage Gel and Decalcified Bone Matrix
Source: Front Cell Dev Biol. 2021 Feb 26;9:638115. doi: 10.3389/fcell.2021.638115 (PMC7952450; doi:10.3389/fcell.2021.638115)

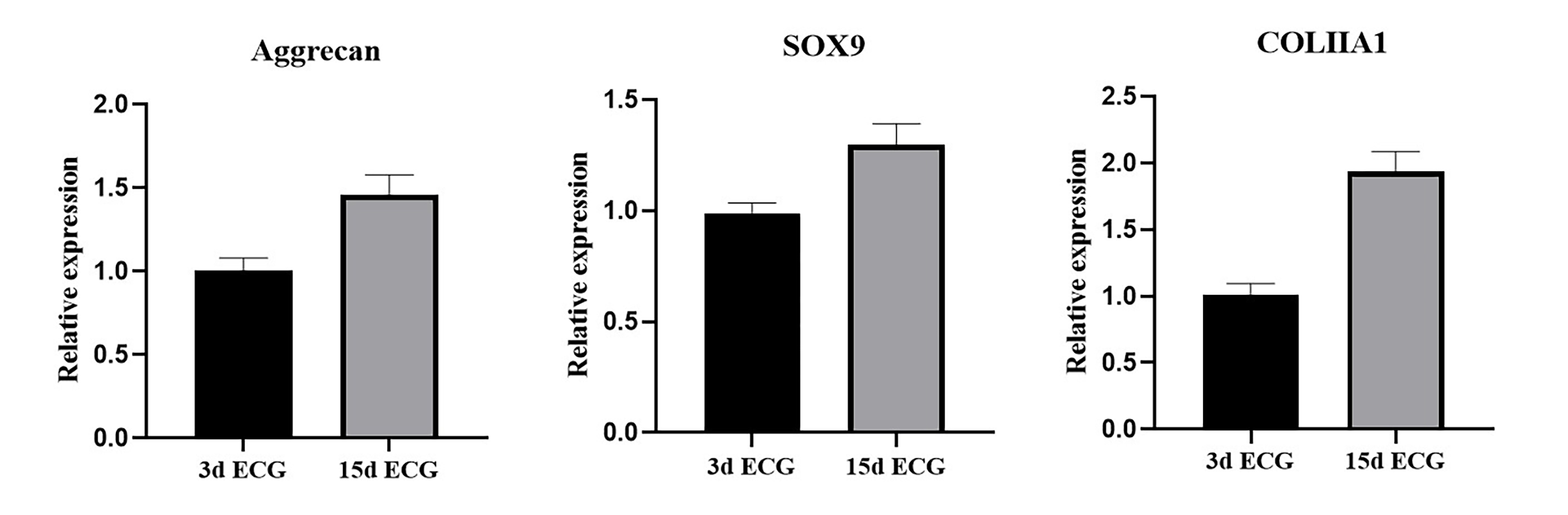

Supplement: Supplementary file 1 [file Image_1.JPEG]
